# Aberrant decision-making as a risk factor for falls in aging

**DOI:** 10.3389/fnagi.2024.1384242

**Published:** 2024-06-24

**Authors:** Shreya Jain, Nicolas Schweighofer, James M. Finley

**Affiliations:** ^1^Division of Biokinesiology and Physical Therapy, University of Southern California, Los Angeles, CA, United States; ^2^Department of Biomedical Engineering, University of Southern California, Los Angeles, CA, United States; ^3^Neuroscience Graduate Program, University of Southern California, Los Angeles, CA, United States

**Keywords:** decision-making, aging, gait, falls, locomotion, risk-taking

## Abstract

Neuromotor impairments resulting from natural aging and aging-related diseases are often accompanied by a heightened prevalence of falls and fall-related injuries. Conventionally, the study of factors contributing to falls focuses on intrinsic characteristics, such as sensorimotor processing delays and weakness, and extrinsic factors, such as environmental obstacles. However, the impact of these factors only becomes evident in response to people’s decisions about how and where they will move in their environment. This decision-making process can be considered a behavioral risk factor, and it influences the extent to which a person engages in activities that place them near the limits of their capacity. While there are readily available tools for assessing intrinsic and extrinsic fall risk, our understanding of how to assess behavioral risk is limited. Measuring behavioral risk requires a systematic assessment of how people make decisions when walking in complex environments and how these decisions relate to their functional capacity. We propose that experimental methods and computational models derived from behavioral economics can stimulate the development of such assessments. Behavioral economics relies on theoretical models and empirical studies to characterize the factors that influence how people make decisions under risky conditions where a given decision can have variable outcomes. Applying a behavioral economic approach to walking can provide insight into how internal assessment of one’s fall risk influences the tasks that one is willing to perform. Ultimately, these assessments will allow us to identify people who make choices that increase their likelihood of fall-related injuries.

## Introduction

Control of bipedal gait is an inherently risky task. As we walk, our body’s center of mass routinely exits our base of support, defined by the contact area between our feet and the ground, and as a result, we must actively control where we place our feet to maintain balance and prevent falls. In the absence of sensorimotor impairments, we learn to maintain balance while walking in various conditions, from smooth, uncluttered environments to uneven, mountainous terrain. This ability to maintain balance while walking is even more impressive when we consider that the nervous system must account for sensorimotor transmission and processing delays (Milton, 2011), signal-dependent noise (Harris and Wolpert, 1998), slow conversion of neural impulses to muscle force (Sandow, 1952), and a high-dimensional action space (Bernshteĭn, 1967). Although the nervous system readily solves the balance control problem despite these challenges, age-related impairments and a variety of neurological and musculoskeletal injuries can dramatically degrade balance and increase fall risk. Several studies over many years have characterized how factors such as slow response times (Smeesters et al., 2001; Pijnappels et al., 2005; Okubo et al., 2017), weakness (Moreland et al., 2004; Ding and Yang, 2016; Lauretani et al., 2018), and impairments in coordination (James et al., 2017; Pozaic et al., 2019; Liu and Finley, 2020) can increase fall risk by limiting people’s ability to respond appropriately to balance perturbations in risky environments. However, much less effort has been devoted to understanding the role of decision-making when people are exposed to situations that may put them at risk of falling.

The purpose of this review is to highlight how the choices people make regarding their future actions influence the likelihood of falls and how age-related changes influence this class of behavioral risk factors. We first highlight differences in how the concept of risk is operationalized in movement science and decision-making, then discuss limitations in contemporary approaches to examine risk-taking behavior during walking. Next, we discuss how theories and methods from behavioral economics can be used to develop models that explain how people make decisions in the context of risk. Finally, we conclude with a perspective of how we can integrate methods from behavioral economics with experimental methods from movement science to understand how age-related changes in the decision-making process may contribute to a heightened risk of falls in older versus younger adults (for key points, see Box 1).

## Managing risk during walking

Risk during walking has classically been conceptualized in two forms within the movement sciences. First, fall risk is commonly considered as the likelihood that a person will experience a fall over a fixed period in the future (Tiedemann et al., 2010). The primary limitation of this definition is that it is not directly observable as there is currently no assessment that precisely predicts how likely someone is to fall over short timescales. Second, risk is often conceptualized with reference to dynamic balance control by characterizing how well people recover from losses of balance to prevent falls during walking (McAndrew et al., 2011; Hak et al., 2012; McAndrew Young et al., 2012; Aprigliano et al., 2016; Liu et al., 2018; Liu and Finley, 2020). For example, it is common for researchers to use biomechanical measures such as dynamic margins of stability (McAndrew Young et al., 2012; Hak et al., 2013; Park and Finley, 2017; Havens et al., 2018; Buurke et al., 2020) and whole-body angular momentum (Herr and Popovic, 2008; Nott et al., 2014; Aprigliano et al., 2016; Liu et al., 2018; Liu and Finley, 2020) to characterize balance control in natural and perturbed gait. Applying perturbations during walking allows researchers to characterize the control strategies used to maintain balance and assess how these strategies differ in people with neuromotor impairments. Although these biomechanical measures are precise, they fail to capture the probabilistic characteristics of behavior associated with the concept of risk.

In contrast to how risk is conceptualized in movement science, risk is formally defined within behavioral economics as the variance of the possible outcomes in a given situation (Markowitz, 1952; Kahneman and Tversky, 1979; Tobler and Weber, 2014). In the context of walking, one can consider a single step to be a form of gamble. Using the definition of risk from behavioral economics, taking steps on a wide path that is free of obstacles would be considered low risk because the probability of losing one’s balance is near zero for people who lack balance impairments. In contrast, walking on an uneven, rocky trail has more variable possible outcomes such as losing balance or falling and hence, has higher risk. What remains to be understood is how people weigh risk when choosing between alternative routes through the environment or when deciding if they should perform a task that may increase their probability of losing balance or falling.

## Behavioral risk

**Figure 1:**
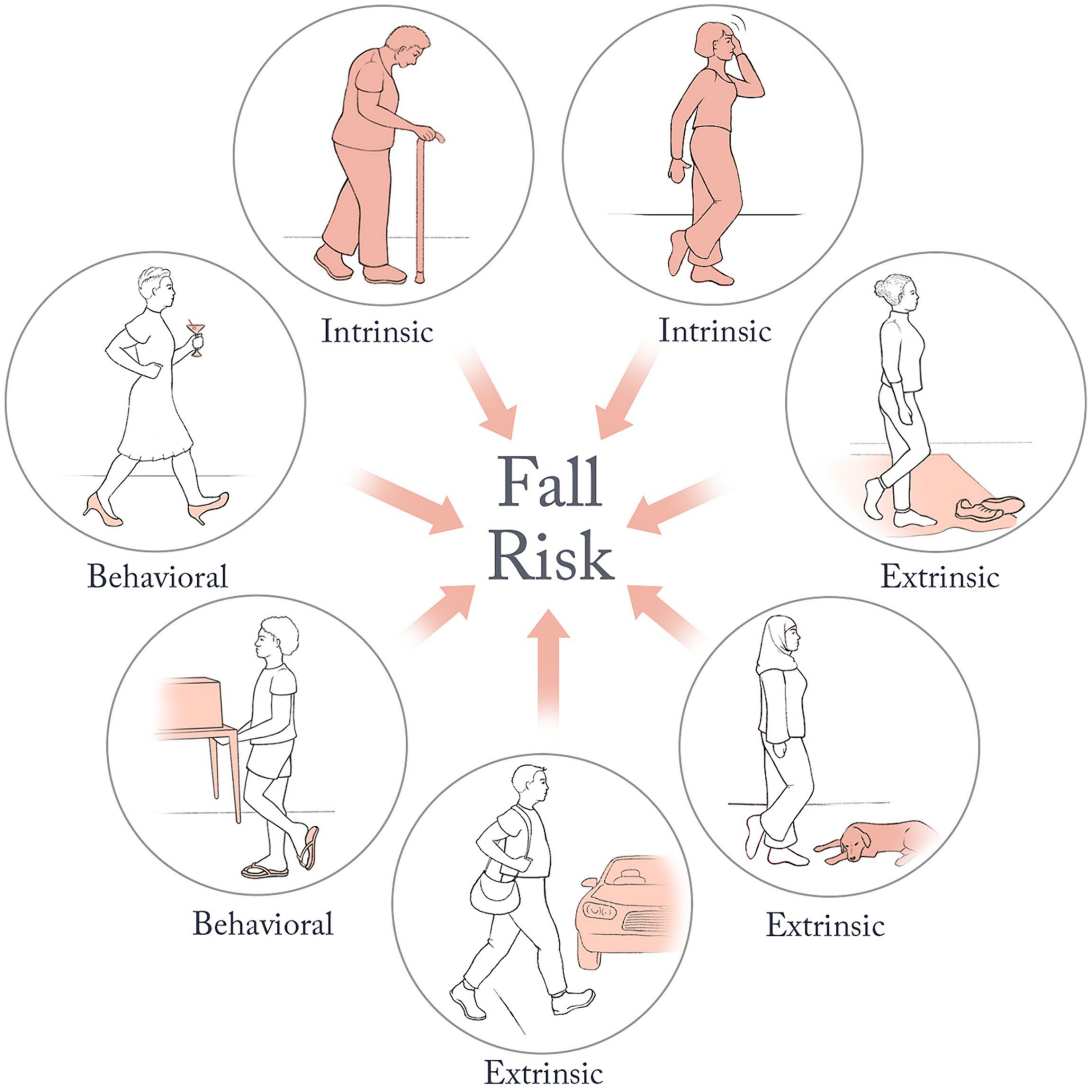
Examples of intrinsic (aging and weakness, dizziness), extrinsic (obstacles, vehicles on the road), and behavioral (carrying heavy objects, walking in heels under the influence of alcohol) factors that influence fall risk.

**Figure 2:**
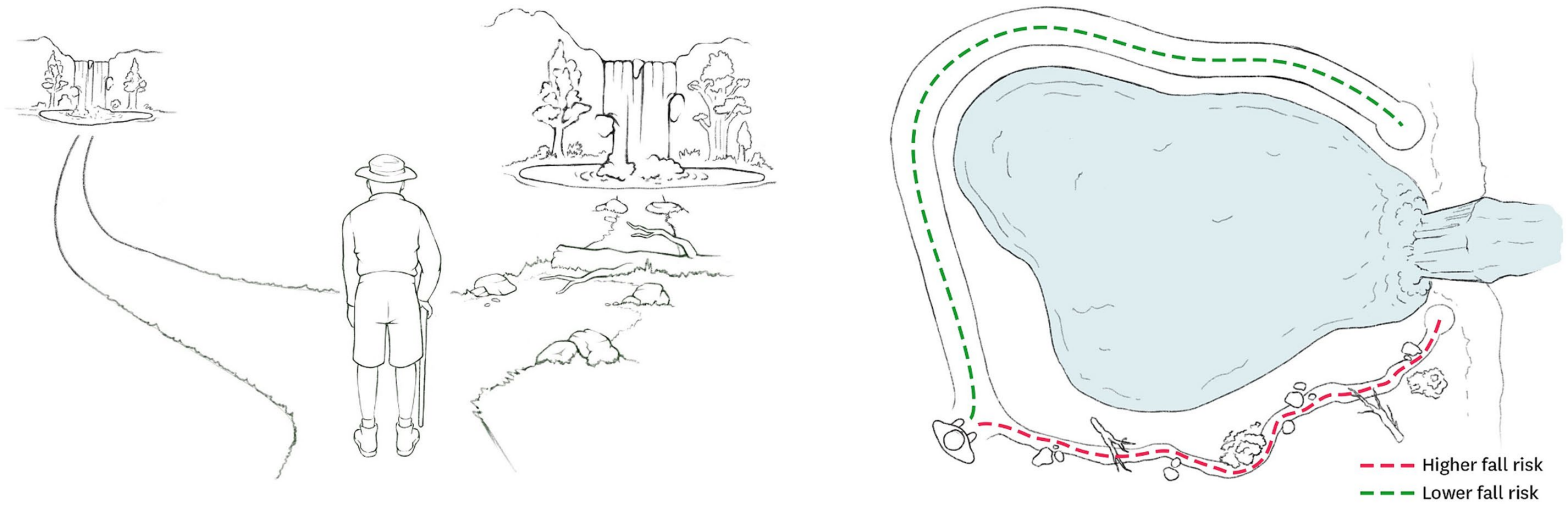
Examples of risky decision-making during walking. Decision between two hiking paths to the same destination—a long path without obstacles (green dashed line) and a short path with several obstacles (red dashed line) such as rocks and fallen trees. (Left) From the perspective of the decision-maker. (Right) Top view of the two options. This scenario represents a decision situation with a trade-off between risk and time such that while the route on the left (or green dashed line) has low risk, it would require more time to reach the destination whereas the opposite is true for the second option. The route on the right (or red dashed line) presents a greater fall and injury risk.

Several factors contribute to the likelihood of an individual falling and these are most commonly divided into three types—intrinsic, extrinsic, and behavioral (Perell et al., 2001; Feldman and Chaudhury, 2008; Figure 1). Intrinsic factors include physical and cognitive characteristics such as sensorimotor processing delays, weakness, gait and balance deficits, and cognitive impairments. Extrinsic factors relate to the presence of hazards in the environment, such as obstacles and slippery surfaces. While these two categories include factors that one can observe and measure, behavioral fall risk is a relatively ill-defined category. Broadly, it relates to people’s choices regarding their actions (WHO, 2007; Feldman and Chaudhury, 2008). These can be directly related to movement, such as hurrying or carrying multiple objects while walking, or they can be indirectly related to movement, such as excessive alcohol use. Another example of a choice that would impact behavioral risk is the decision one makes when faced with multiple candidate paths linking one location to another. For example, when hiking through a forest, one might encounter two potential routes leading to the same destination: a long but smooth path and a short but uneven path with loose gravel and obstacles (Figure 2). Deciding between these routes requires a person to estimate the riskiness of each option based on estimates of their capacity and properties of the environment, and manage a trade-off between this perceived risk, time, and effort. This decision-making process is particularly critical for elderly individuals who are at risk of catastrophic injury from falls. Aging is often accompanied by cognitive impairments in domains such as executive function, working memory, and fluid intelligence, which may also influence decision-making (Del Missier et al., 2012; Brand and Schiebener, 2013; Tymula et al., 2013; Trevisan et al., 2019; Waltrip et al., 2023).

Age-dependent changes in neurobiological processes can increase falls through their effects on decision-making. Decision-making is largely attributed to frontal areas of the brain such as the posterior parietal cortex and lateral prefrontal cortex, which show reductions in gray matter volume with aging (Resnick et al., 2003; Driscoll et al., 2009; Kennedy et al., 2009). In addition, dopamine plays a key role in assigning subjective values to different choice options in risky decision-making (Levy et al., 2010), and dopamine receptor availability in the prefrontal cortex has been shown to decline with aging (Karrer et al., 2017). It has been proposed that these declines lead to decisions made more from emotional or affect-based processes than from analytical processes (Lighthall, 2020). Additionally, the cholinergic system has been implicated in falls through its role in attentional focus such that older adults who fall have lower cholinergic activity than those who do not and among the fallers, the cholinergic activity is associated with gait speed (Pelosin et al., 2016). This is particularly interesting because of the role that inattention plays in promoting impulsivity in older adults during gait, thereby increasing fall risk (Harrison et al., 2010; Ferrari et al., 2012). Impulsivity refers to making decisions without considering the outcomes (Harrison et al., 2010) or failing to consider the immediate environment and safety concerns when moving (Ferrari et al., 2012) and therefore, has a direct impact on behavioral fall risk. Overall, these results provide a neurophysiological basis for a complex interplay between motor impairments, cognitive decline, and decision-making with aging, which may result in elevated fall risk in older adults.

While some studies and reviews allude to the presence of behavioral risk factors, these factors have historically been understudied. A multifactorial framework for fall risk has been proposed based on a review of 25 studies investigating relationships between features of the physical environment and falls in older adults (Feldman and Chaudhury, 2008). Three main fall risk factors were identified, the interactions between which determine the risk of falls: mobility, features of the physical environment, and risk-taking behavior. Mobility was defined as the individual’s ability to perform movements, which can be affected by aging, weakness, and disorders such as Parkinson’s disease and stroke. Features of the physical environment included the presence of hazards or safety features in people’s homes and daily use environments, for example, grab bars in the bathroom or railings on staircases. Risk-taking behaviors were defined as those that increase the likelihood of falling or those that challenge an individual’s dynamic balance. Thus, risk-taking behaviors are specific to each individual, such that a behavior that may be risk-taking for one individual might not be for another. Inappropriate risk-taking behaviors are reported as being a major cause of falls among older adults (Connell and Wolf, 1997; Feldman and Chaudhury, 2008). This fall risk framework, however, does not propose a method to objectively assess risk-taking.

### Self-reported assessments of behavioral risk

Because an individual’s risk-taking behaviors are not easily observed, clinical assessments instead capture people’s perceptions of their ability to maintain balance in risky environments using scales such as the Falls Efficacy Scale, Activities-Specific Balance Confidence Scale, and the Modified Gait Efficacy Scale (Delbaere et al., 2010; Hadjistavropoulos et al., 2011; Moreira et al., 2017; Kluft et al., 2020). The Falls Efficacy Scale assesses the fear of falling by asking respondents to rate their level of concern about falling while performing different activities. The Activities-Specific Balance Confidence Scale and the Modified Gait Efficacy Scale measure balance confidence by having respondents rate their level of confidence in their ability to perform different activities without losing their balance. Though neither falls efficacy nor balance confidence assess decision-making, they can be used to gain insight into risk-seeking tendencies such that individuals with high falls efficacy and high balance confidence may be more willing to take risk, regardless of their actual physical ability. The Falls Behavioral Scale for Older People assesses people’s *perceptions* of their behaviors when faced with risky situations (Clemson et al., 2003). Respondents can rate the frequency with which they engage in protective behaviors, such as holding on to a handrail when using stairs and using a walking aid when needed, and risky behaviors, such as hurrying when doing things. Each of these questionnaires relies on self-reports and, hence, necessarily assesses people’s perceptions of their behaviors and not their actual behaviors. These types of questionnaires are also subject to self-reporting biases (Raphael, 1987; Grimm, 2010; Althubaiti, 2016), including recall and social desirability such that people may tend toward reporting their behaviors as being more cautious than they actually are.

### Experimental methods of assessing behavioral risk while walking

Plank-crossing is a walking task that has been used to assess behavioral risk in older adults, such that when participants are free to choose from planks of different widths and heights to walk across, their choice can indicate their level of behavioral risk (Butler et al., 2015). Older adults who took higher behavioral risk in this task self-reported as being cautious in their everyday lives (Butler et al., 2015). This mismatch could potentially be due to the previously discussed self-reporting biases, incorrect estimates of gait ability during plank-crossing, impaired sensorimotor integration or cognitive deficits (Li and Lindenberger, 2002). These same older adults also experienced more falls in a 12-month follow-up period. Additionally, there was no association between precision walking ability and behavioral risk as those with better ability chose lower-risk planks and vice versa. One limitation of this study, however, is that plank crossing speed was not controlled or accounted for and can itself be a source of risk due to its influence on foot placement accuracy (Bradshaw and Sparrow, 2000; Roerdink et al., 2021). A second limitation is that participants did not cross the chosen planks, and thus the chosen speed of plank-crossing and the likelihood of making a misstep were unknown.

A mismatch has also been found between reported risk-taking during road-crossing and actual observed road-crossing decisions in older adults (Butler et al., 2016). Road-crossing is a common risky activity that requires a good estimate of one’s ability and accurate perceptions of the speed of moving vehicles. In a study of road-crossing decisions, a simulated pedestrian crossing was created and participants were instructed to cross in front of a styrofoam car, at the shortest possible distance from it (Butler et al., 2016). One group crossed within a small distance from the car, suggesting that they accurately judged their ability and their environment. This group also performed best on a battery of physical and cognitive tests. However, they reported engaging in risky behavior and being less cautious in everyday life. Conversely, the groups that were either “hit” by the car or had to retreat to avoid being “hit” reported being less risky and more cautious on the everyday risk-taking scale.

A major limitation of the studies described above was that only a single decision was made in each condition. In everyday life, we tend to face similar decision-making situations multiple times and our decisions generally change over time as we learn from experience. It is important to understand how experience influences decision-making and whether experience-dependent changes are appropriate, given the individual’s motor ability. These studies were also limited in the range of risky conditions that could safely be used. One potential way to address this limitation is to use virtual reality (VR) in combination with physical perturbations to create complex walking scenarios that mimic the real world (Cano Porras et al., 2018, 2019; Raffegeau et al., 2023). An advantage of this method is that it would allow for the evaluation of decision-making in real-world scenarios within a controlled and safe environment. Using physical perturbations delivered via motion platforms or specialized treadmills can further improve ecological validity by introducing balance-disturbing consequences to decisions that may better reflect what people experience in the real world (Park and Finley, 2017; Lee et al., 2019; Buurke et al., 2020; Debelle et al., 2020). This would allow researchers to systematically capture the effects of experience, the visual representation of risk, and the physical consequences of errors on decision-making while walking.

### The role of misjudgment in risky decision-making during walking

The results above suggest a mismatch between people’s perception and memory of how they manage risk while walking and their behaviors. Similarly, there is a mismatch between perceived and actual motor ability. In older adults, it is possible that a decline in physical function with aging may not always be accompanied by a perception of this decline (Butler et al., 2016). In risky situations, this mismatch between perceived and actual ability has the potential to increase the probability of a fall. For example, if an individual who overestimates their stepping ability faces an obstacle on a hiking trail, they may incorrectly choose to step over it instead of walking around it, thereby putting themselves at an increased risk of tripping or falling and injuring themselves. Therefore, there is a need to investigate this construct of “misjudgment” between perceived and actual ability and its contribution to fall risk.

In general, misjudgment is quantified by measuring perceived ability through an oral response and actual ability through the performance of the task. Studies of misjudgment in motor tasks are summarized in Table 1. One example of such a task is precision walking wherein perceived ability was measured by asking participants to estimate the narrowest path within which they could walk without stepping outside its boundaries (Kluft et al., 2017). Actual ability was measured by determining the actual narrowest path within which they could accurately walk. The degree of misjudgment in this paradigm was found to be highly variable among participants, with many older adults overestimating their walking ability (Lighthall, 2020). Additionally, older adults with a better precision walking ability were not better judges of their accuracy than those with a lower ability. Therefore, judgment of ability appears to be a separate skill in older adults, independent of one’s actual ability.

**Table 1:** Summary of studies of misjudgement of ability in different gait-related motor tasks.

**Type of motor ability to be judged**	**Illustration**	**Key findings**
Maximum forward reach distance (Butler et al., 2011)	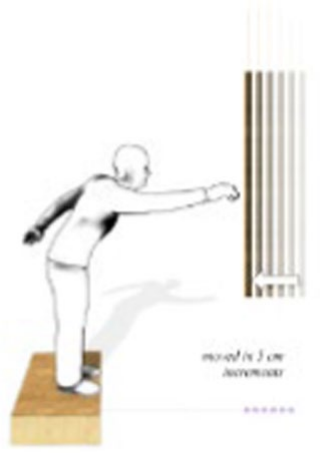	1. No association between differences in estimated and actual maximum reach distance and falls over one year, both retrospective and prospective 2. Greater reach ability associated with lower judgement error
Maximum step-over height (Sakurai et al., 2013)	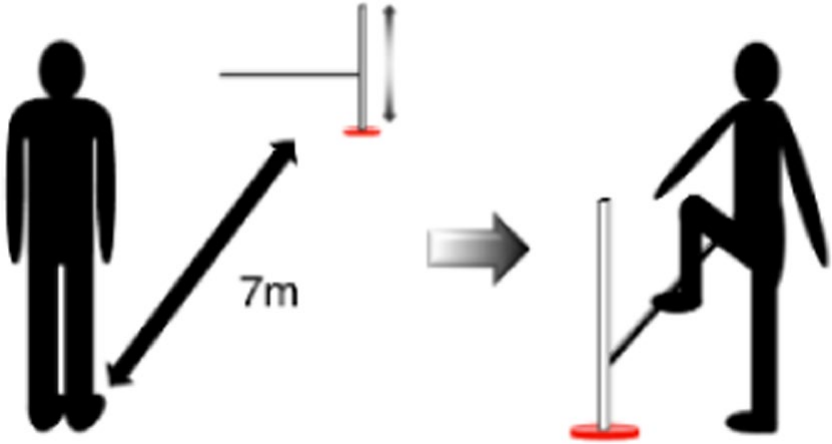	1. No correlation between actual measured maximum step-over height and the perceived maximum height for older adults 2. Greater overestimation of ability among fallers
Narrowest plank that can be crossed quickly without falling (Butler et al., 2015)	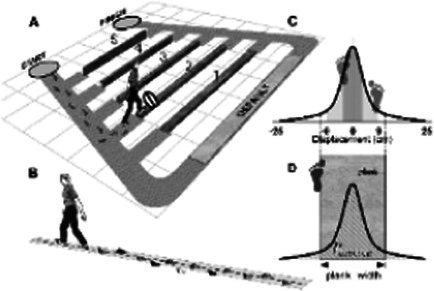	1. Those who chose riskier planks, whose widths were narrower than that required to cross successfully, reported cautious everyday behavior. 2. Level of risk in plank choice, measured as the probability of falling off the chosen plank, was a significant predictor of falls.
Ability to cross a simulated road while leaving a short final gap from a simulated moving car (Butler et al., 2016)	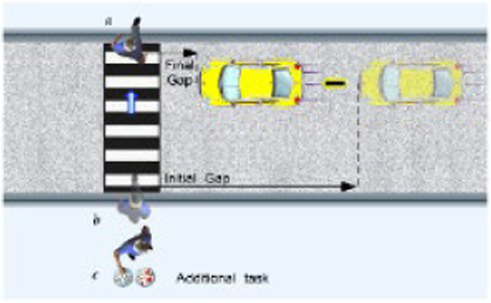	1. Exact crossing decisions leaving a small gap from the car made by those with better performance on a series of physiological and cognitive tests 2. Those who made unsafe crossing decisions, leading to being ‘hit’ or having to retreat to avoid being ‘hit’, reported cautious everyday behavior.
Accuracy of foot placement inside a projected path of varying width for different gait speeds (Kluft et al., 2017b)	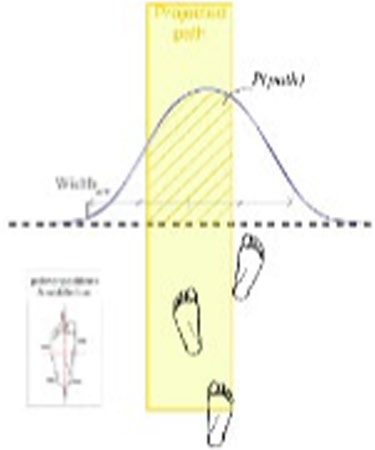	1. No association between actual ability to stay within the bounds of a narrow path and perceived ability 2. Degree of misjudgement between actual and perceived ability not associated with actual ability
Maximum step-over height; Maximum forward stepping distance; Maximum forward lean angle (Kluft et al., 2017a)	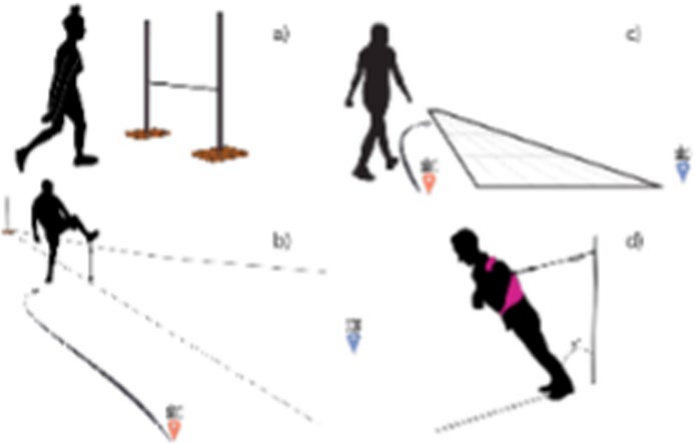	1. Significant correlation between perceived and actual ability in all but the forward lean task 2. No consistency in the extent of misjudgement across the four judgement tasks
Maximum stepping down height using a heel-first strategy (Kluft et al., 2018)	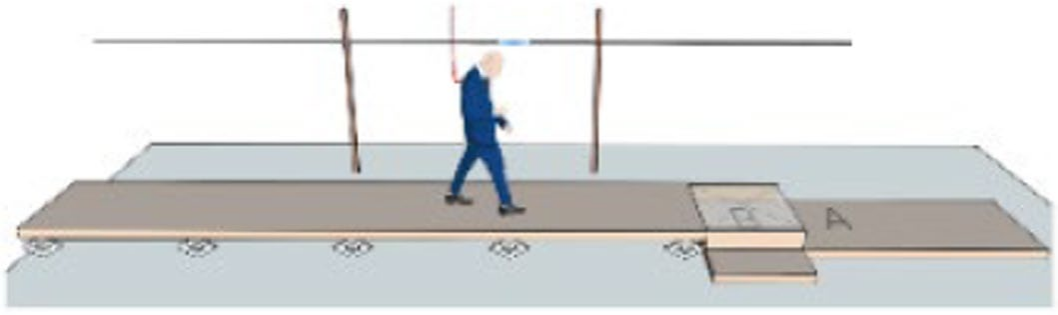	Perceived ability, measured as the step height at which there was an equal probability of choosing a heel-first and toe-first strategy, was not associated with actual ability, measured as the ability to recover balance from an unexpected step-down.
Maximum stepping down height using a heel-first strategy (Kluft et al., 2019)	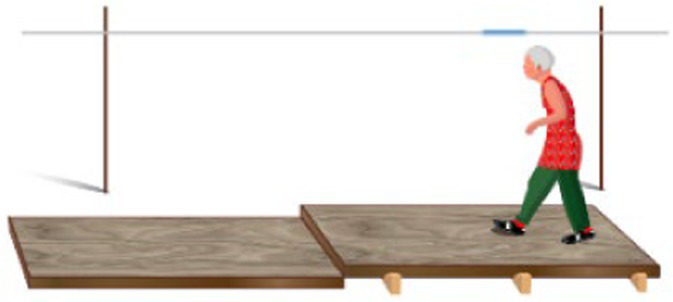	A fall prediction model that included a misjudgement term between actual stepping ability (maximum step height and step length) and perceived ability (step height with an equal probability of heel-first and toe-first strategy) did not perform better when predicting falls over a 10-month period than a model without the misjudgement term.

The table briefly describes the type of motor task studied and the key findings with respect to judgement of ability. Illustrations of the tasks are included. Butler, Annie A.; Lord, Stephen R., Reach Distance but Not Judgment Error Is Associated With Falls in Older People, Journals of Gerontology - Series A: Biological Sciences and Medical Sciences, 2011, 66A, 8, by permission of Oxford University Press. Butler, Annie A.; Lord, Stephen R., Ability Versus Hazard: Risk-Taking and Falls in Older People, Journals of Gerontology - Series A: Biological Sciences and Medical Sciences, 2014, 70, 5, by permission of Oxford University Press. Kluft, Nick; van Dieën, Jaap H.; Pijnappels, Mirjam., The degree of misjudgment between perceived and actual gait ability in older adults, Gait & Posture, 2017, 51 with permission Elsevier. All other images are distributed under the terms of the Creative Commons Attribution License.

Preliminary evidence suggests that misjudgment of ability is task-specific and not an inherent trait. Misjudgment was quantified across four different stepping tasks that involved stepping over a height, stepping across a certain distance, or taking a step to recover from a forward lean (Kluft et al., 2017). There was no association between the degrees of misjudgment across these tasks, suggesting that this may not be a trait inherent to an individual but rather a task-specific skill. While further work has been done to develop more tasks to better assess misjudgment, a good method for quantification of misjudgment has not yet been identified (Kluft et al., 2018).

## Decision-making under risk

While judgment of ability is one factor in walking-related behavioral decisions, these are complex decisions that require an evaluation of the walking environment and the possible outcomes. As a result, there is a need to develop a quantitative framework to better explain how people integrate information about themselves and the environment to inform their decisions. Behavioral economics is a field of study that investigates the psychological and cognitive processes underlying decision-making (Glimcher and Fehr, 2014). Theories in this field provide a framework to study people’s choices in different contexts and the underlying processes that lead to these choices. In the context of behavioral economics, a risky prospect is defined as a situation in which the possible outcome of choosing a specific option is uncertain, and risk is defined as the variance in the distribution of possible outcomes (Braun et al., 2011; Nagengast et al., 2011).

Models of risky decision-making typically maximize a utility function. For example, the mean–variance model expresses the utility of an option, U(x), as a function of the expected value of the possible outcomes, E(x), and their variance (Equation 1) (Markowitz, 1952; Nagengast et al., 2011).


(1) $$U\left(x\right)=E\left(x\right)-\mathrm{\theta Var}\left(x\right)$$

Here, *θ* is the risk-sensitivity parameter where a value of zero indicates risk neutrality, a positive value indicates risk aversion, and a negative value indicates a risk-seeking tendency. This model has previously been applied in effort-based decision-making, in which the “sure bet” required the exertion of a known fixed force by the hand, whereas the risky option could require the individual to exert a lower or higher force than the fixed option, thereby having a larger variance (Nagengast et al., 2011). This and other studies that have applied behavioral economic models of decision-making to motor control are summarized in Table 2. Fitting this model to the participants’ behavior demonstrated that they were sensitive to the level of risk and had tendencies to choose riskier options.

**Table 2:** Summary of studies examining the influence of risk on decision-making in motor tasks.

**Type of task**	**Goal of decision-making task**	**Illustration of task (if available)**	**Decision-making models applied**	**Key findings**
Reach and point to a reward circle on a screen, partially overlapping with a penalty circle (Trommershäuser et al., 2003a)	Maximize accumulated reward points	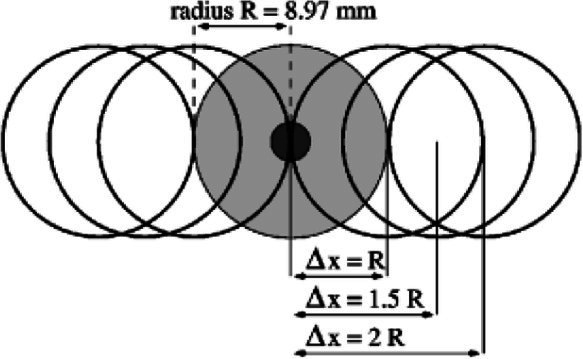	**Statistical Decision Theory** Endpoint (x,y) minimizes: $L\left(x,y\right)=C_{o}P\left(x,y\right)+C_{1}P\left(R_{1}|,x,y\right)$ *C_o_*: Target reward, *R_o_*: Target region, *C_1_*: Penalty points, *R_1_*: Penalty region	1. Optimal endpoints predicted by the model matched actual endpoints. 2. Endpoints shifted away from penalty circle as penalty increased and as penalty circle moved closer to target circle.
Make choices between equivalent economic and motor lotteries (Wu et al., 2009)	Maximize reward accumulated in both types of decision-making tasks	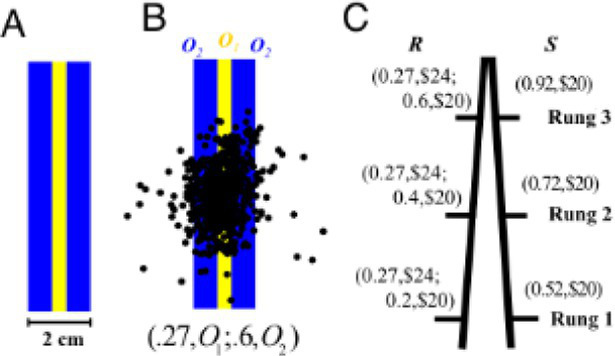	**Prospect Theory** Value function for outcome O: $v\left(O\right)=\{O^{\alpha },O\geq 0-{\left(-O\right)}^{\beta },\;O<0$ Probability (*p*) weighting function: $w\left(p\right)=\exp\left[-{\left(-\ln\;\ln\;\left(p\right)\;\right)}^{\gamma }\right]$	1. Risky lotteries chosen with higher frequency in motor decision-making 2. Overweighting of small and underweighting of large probabilities in economic task; vice-versa in motor task
Make choices between motor lotteries whose outcomes are forces to be exerted on a handheld manipulandum (Nagengast et al., 2011)	Not applicable	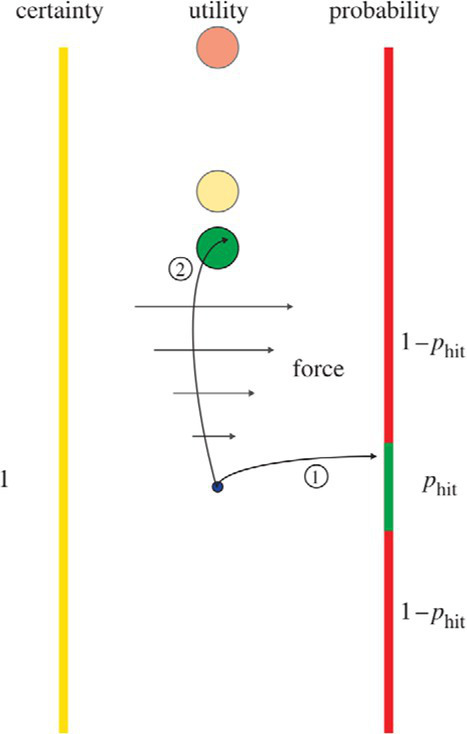	**Mean-Variance Model** For an option with possible force exertions *x*: $U=-E\left(x\right)+\theta *Var\left(x\right)$ *E(x)*: Mean required force, *Var(x)*: Variance of required force, θ : Risk-attitude parameter **Cumulative Prospect Theory** For an option with possible force exertion *x* with probabilities *p*: $v\left(x\right)=-x^{\alpha }$ $w\left(p\right)=\exp\left[-{\left(-\ln\;\ln\;p\right)}^{\gamma }\right]$	1. Sensitivity to risk was present, in the direction of risk-seeking. 2. Mean-Variance model was a better fit to the data than Cumulative Prospect Theory.
Movement of handheld manipulandum to control the position of a cursor under different noise conditions and end at the center of a target line (Nagengast et al., 2010)	Minimize final target error (error cost) and the amount of control applied to the manipulandum (control cost)	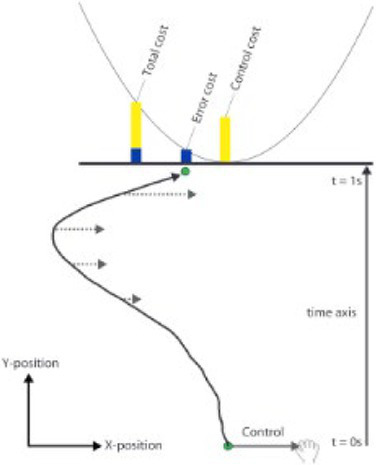	**Mean-Variance Model** Given a cost function C, an optimal controller minimizes: $E\left(C\right)-\theta *Var\left(C\right)$ E(C): Mean of cost function, Var(C): Variance of cost function, θ : Risk-sensitivity parameter	1. Sensitivity to risk was present, in the direction of risk-aversion. 2. When noise was high, participants were willing to incur a control cost to avoid movement errors.
Movement of handheld manipulandum to control the position of a cursor from a start to end position and passing through a target region, under different levels of visual feedback uncertainty (Grau-Moya et al., 2012)	Minimize target error and movement cost, explicitly added in the form of horizontal viscous force	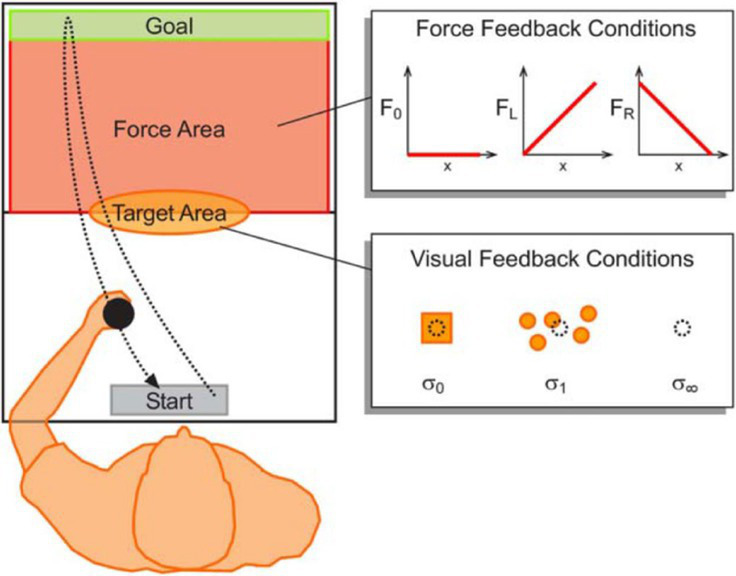	**Risk-Sensitive Bayesian Integration** Optimize the stress function given by: $u^{\mathrm{opt}}=\frac{\sigma_{p}^{2}}{\sigma_{i}^{2}+\sigma_{p}^{2}}y-\frac{a_{j}}{2Q}-\frac{\sigma_{i}^{2}\sigma_{p}^{2}}{\sigma_{i}^{2}+\sigma_{p}^{2}}\theta a_{j}$ : target position, $\sigma_{p}$ : Uncertainty in visual feedback, *y*: Observed target position, $\sigma_{i}$ : Strength of viscous force, *Q*: importance of reaching the target, $a_{j}$ : risk-sensitivity parameter	1. Decisions were based on both, feedback uncertainty and movement cost. 2. As uncertainty increased, movements were more biased towards low movement cost regions.
Swift out and back movements with the hand and whole-body lean to control the position of a cursor under different levels of noise (O’Brien et al., 2013)	Maximize reward points by moving greater distances but avoiding a penalty region at the end	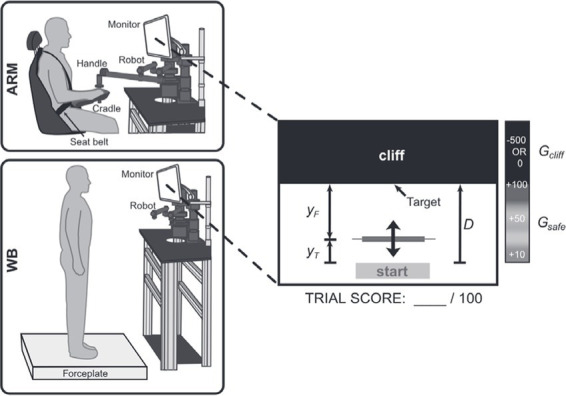	**Statistical Decision Theory** Expected gain function for a chosen movement strategy *y*: $\Gamma \left(y\right)=\{G_{\mathrm{safe}}P\left(y\right)\;\mathrm{if}\;y^{\prime }\leq y_{\mathrm{cliff}}\;G_{\mathrm{cliff}}P\left(y\right)\;\mathrm{if}\;y^{\prime }>y_{\mathrm{cliff}}$ *G_i_*: Gain associate with region, *y*: Planned endpoint, *y’*: Actual endpoint **Cumulative Prospect Theory** Expected gain function for a chosen movement strategy *y*: $\Gamma \left(y\right)=\{G_{i}^{\alpha }w\left(P\right),\;\mathrm{if}\;G_{i}\geq 0-{\left(-G_{i}\right)}^{\beta }w\left(P\right),\;\mathrm{if}\;G_{i}>0$ $w\left(P\right)=\exp\left\{-{\left[-\mathrm{logP}\left(y\right)\right]}^{\gamma }\right\}$	1. Sensitivity to risk was present, in the direction of risk-seeking, for both types of movements. 2. Higher risk-seeking in the whole-body movement.

For each study, the type of task, the task objective, an illustration of the task, a list of the decision-making models used, and the key findings are listed. Maloney, Laurence T.; Landy, Michael S. Statistical decision theory and trade-offs in the control of motor response, Spatial Vision, 2003, 16(3-4), 255. Reprinted with permission from Spatial Vision © Optical Society of America. Copyright (2009) National Academy of Sciences. O’Brien, Megan K.; Ahmed, Alaa A., Does risk-sensitivity transfer across movements?, Journal of Neurophysiology, 2013, 109, 7 with permission Elsevier. All other images are distributed under the terms of the Creative Commons Attribution License.

A model of motor decision-making has been proposed that accounts for the inherent uncertainty in movement planning and execution in addition to biomechanical costs such as effort (Trommershäuser et al., 2003a). To test this model, partially overlapping target and penalty areas were presented on a screen. A reaching movement that ended in the target circle yielded reward points, whereas the penalty circle led to a loss. A prediction was made for each individual’s optimal movement endpoint that would maximize reward while accounting for their natural movement variability. This prediction was compared to their actual chosen endpoints. Actual decisions closely matched those of an “optimal performer” suggesting that people accurately take both their own movement variability or uncertainty as well as explicit costs into account when making decisions for such pointing tasks (Trommershäuser et al., 2003a).

Prospect Theory is a model of risky decision-making that was developed to explain commonly observed “irrationalities” in people’s behaviors (Kahneman and Tversky, 1979; Tversky and Kahneman, 1992). These include risk-seeking tendencies in the presence of small probability gains (e.g., gambling), and risk-aversive tendencies in the presence of small probability losses (e.g., purchasing insurance). According to this model, people’s choices can be explained by a process in which they transform and represent the probability and values of options available to them and use these representations to make a decision (Kahneman and Tversky, 1979; Tversky and Kahneman, 1992). Prospect Theory considers three key elements of decision-making: diminishing sensitivity, loss aversion, and probability distortion. For an option with a possible outcome *x* that has a probability *p*, Prospect Theory defines its subjective value *v(x)* and decision weight *w(p)* as in the following equations.


(2) $$v\left(x\right)=\{\begin{array}{c} x^{\alpha }\;for\;x\geq 0\\ -\lambda {\left(-x\right)}^{\beta }\;for\;x<0\; \end{array}$$

(3) $$w\left(p\right)=\exp\left[-{\left(-\ln p\right)}^{\gamma }\right]$$

Consider a lottery that offers $50 with probability *p* and $0 with probability 1-*p*. First, Prospect Theory suggests that the subjective value of a gain of $50 is higher for someone who only has $100 than for someone with $10,000. This feature reflects a value function that has a diminishing sensitivity to change (Equation 2, when *ɑ* < 1 or 𝛽 < 1). Second, the theory captures loss aversion for which the value function is steeper for losses than for gains. This leads to losses having a higher impact on value than a gain of equivalent magnitude (𝜆 in Equation 2). Third, the theory characterizes probability distortion, according to which people do not objectively represent the probabilities of possible outcomes in a lottery. Instead, they transform the probabilities to decision weights, w(p), that determine the impact of each outcome on the person’s decision (Equation 3). This distortion function is generally represented by a sigmoidal function, which is steepest near probabilities of 0 and 1 (Tversky and Kahneman, 1992). A more recent version of this theory, called Cumulative Prospect Theory, applies the probability weighting function to cumulative probabilities of outcomes and is generalizable to decisions that include more than two possible outcomes (Tversky and Kahneman, 1992).

Several studies have used the Prospect Theory framework to investigate motor decision-making, subjective valuation, and probability distortion during motor tasks (Wu et al., 2009; O’Brien and Ahmed, 2013; O’Brien and Ahmed, 2014; O’Brien and Ahmed, 2015). A comparison between equivalent economic and motor decision-making tasks observed typical overweighting of small probabilities and underweighting of moderate to large probabilities in the economic domain but the opposite tendency in the motor domain (Wu et al., 2009). “Motor lotteries” were presented in the form of targets of varying widths, which, combined with each individual’s motor variability, determined the probability of success of a reaching movement. Each target was associated with some reward equivalent to those in the economic task. Therefore, with all else being equal, the only difference between the two tasks was the manner in which probability information was provided—explicitly in the economic task and implicitly based on the target width in the motor task. These results suggest that the probability weighting function depends on the task, the nature in which probability information is provided, or both (Box 2).

Within the motor domain, risk-seeking has been observed in both upper-body reaching and whole-body leaning type of movements (O’Brien and Ahmed, 2013). Similar to previously reported results, underweighting of small and overweighting of moderate to large probabilities was observed in both these movements, along with the overvaluation of rewards and undervaluation of penalties (O’Brien and Ahmed, 2013). In both tasks, swift out-and-back movements were performed to control a cursor on a screen using either a manipulandum for the reaching task or by leaning forward on a force plate for the whole-body task. The cursor was taken from its home position to as close as possible to the edge of a virtual “cliff.” Risk was manipulated by either varying the penalty associated with the cliff region or by adding noise to the cursor position. Surprisingly, risk-seeking was greater in the whole-body task than in the arm-reaching task. This result is counterintuitive because one would expect that a whole-body movement, which has a more unstable posture than a seated reaching movement, might lead to risk-aversive tendencies. However, it is important to note that the cliff and the consequences of risk-taking in this task are artificial, and therefore, these results may not generalize to the presence of real, physical consequences.

When the same two tasks described above were performed on a platform at a height of 0.8 m above the ground, the difference in the extent of risk-seeking between the two was eliminated (O’Brien and Ahmed, 2015). Decision analysis using CPT revealed a significant difference in probability weighting in the whole-body movement between the two elevations, such that small probability losses were overestimated to a greater extent at high elevations, leading to more risk-averse behavior. Therefore, it seems that implicit postural threat increases risk sensitivity in the context of movement-related decisions. While these two studies were the first to assess risk-sensitive decision-making in goal-directed whole-body movements using a behavioral economic approach, more work remains to be done using tasks with actual physical consequences to understand how the perception of risk and its effects on decision-making is affected by movement type, context, and experience.

While these studies have considerably advanced the understanding of motor decision-making in young adults, the effects of aging in this decision domain are not well studied. There is some evidence of decreased risk-taking in older adults compared to young adults in a reach-to-target task where risk is manipulated using penalty points (Valsecchi et al., 2018). In a plank-crossing task described in a previous section, mixed results were reported among older adults with some choosing to cross risky planks and some choosing safer options (Butler et al., 2015). However, this study did not compare young and older adults, making it difficult to ascertain whether risk-taking in this task is age-dependent. Studies comparing decision-making across multiple domains suggest an interaction between domain and age such that risk-seeking generally decreases with age in the financial, health, ethical, and recreational domains but might increase with age in the social domain (Weber et al., 2002; Blais and Weber, 2006; Rolison et al., 2014; Waltrip et al., 2023). More work is needed, specifically in the context of gait-related decision-making, to understand how aging influences behavioral fall risk.

## Opportunities for advancing theoretical understanding and assessment of behavioral risk during walking

As reviewed above, fall risk assessments in the clinic largely focus on identifying intrinsic and extrinsic fall risk factors, often rely on self-reports, and do not have a high predictive value for falls (Lee et al., 2013). One potential reason for the limited predictive ability of current assessments is that they neglect an assessment of actual decisions in the context of risk. The emergence of intrinsic risk factors with age or disease is likely accompanied by changes in decisions that people make. For example, a decline in visual acuity may lead to an individual consciously adopting more cautious behaviors such as walking slower. Conversely, the lack of intrinsic or extrinsic risk factors is not necessarily indicative of low fall risk as the individual’s behavioral decisions could be putting them in risky situations. Therefore, this interplay between intrinsic and extrinsic risk factors and behavioral decisions needs to be investigated, and this requires the means to systematically assess people’s motor decision-making in risky environments.

The need to assess behavioral risk has been addressed in part by developing self-report questionnaires that ask respondents to rate their level of engagement in risky behaviors in everyday life (Clemson et al., 2003; Kwan et al., 2013; Butler et al., 2015). However, such questionnaires rely on people’s perceptions and memories of their actions and can, therefore be subject to common biases such as recall bias and social desirability bias (Althubaiti, 2016). In fact, as reviewed above, people’s self-reports of risky behaviors in everyday life do not always match the nature of their actual decisions in behavioral tasks (Butler et al., 2015, 2016). This points toward the need to assess people’s actual decision-making during walking, the factors that influence this decision-making process, and the relationship between walking decisions and fall risk.

We propose that experimental methods and computational models derived from behavioral economics can overcome the limitations associated with assessments of behavioral risk that are based primarily on self-reporting. A behavioral economic approach can be used to identify the cognitive processes that underlie decision-making in complex environments and identify individuals who may be at a higher risk of falls because they engage in risk-seeking behaviors. The Mean–Variance model and Prospect Theory are examples of integrative or algebraic models of decision-making, which assume that people assess all the information about all available options before making a decision. While these models have been fairly successful in explaining people’s economic decisions, they are not considered to be plausible representations of the underlying cognitive computations that mediate decision-making (Payne et al., 1978; Pachur et al., 2017). For example, when we decide between alternate paths along a rocky trail, we do not have explicit information about the probabilities of different outcomes (e.g., losses of balance) associated with each path. Even in situations where all this information is explicitly available, the ability to perform a Prospect Theory-like algebraic analysis may not be psychologically plausible (Payne et al., 1978; Lopes, 1995; Pachur et al., 2017).

To this end, heuristic models of decision-making have been proposed as more plausible strategies by which people make decisions. Individuals with high levels of statistical numeracy have been shown to use heuristic rather than algebraic models of decision-making, which leads to more consistent choices between risky economic gambles (Ashby, 2017). These models assume that people have a limited capacity for memory and information processing and as a result, filter the available information to simplify the decision-making process (Slovic et al., 2004; Gonzalez and Mehlhorn, 2016; Pachur et al., 2017). An example of this filtering process is the recency bias, according to which people more heavily weigh events that were experienced closer in time to the decision (Hertwig and Erev, 2009). In the context of gait, if a decision about a walking path must be made quickly, this heuristic may be utilized and allow for more recent experiences of losses of balance to influence the decision. Another heuristic, which is particularly relevant in the context of walking and falls, is the affect heuristic (Slovic et al., 2004). Affect is the feeling of positivity or negativity that we associate with any event or object. By associating each of our experiences with a general “feeling” or “affect,” we can perform a quick analysis of possible outcomes of a decision in the future by identifying the affect associated with each. This becomes particularly relevant when studying motor decisions that involve whole-body movements because of the affective response that near-falls or falls elicit. The use of heuristic processes in simple choices has also been shown to lead to more consistent decisions than in complex choices with higher cognitive demands (Bessette et al., 2021). However, the extent to which the use of more cognitively demanding deliberative and simpler heuristic decision-making processes explains individual differences in behavioral risk during walking remains to be seen.

The development of a comprehensive framework of decision-making during walking requires an understanding of how different factors, such as risk or uncertainty, time, movement goals, movement context, and personality traits, influence decisions. Future studies should aim to study these factors in isolation and in combination to best approximate people’s decision-making behaviors in more complex, real-world situations. The influence of risk or uncertainty can be determined by having people choose between walking options with different levels of variability in the possible outcomes of each. These outcomes may be presented in the form of trips or slips, which are often the cause of falls in both young and older adults and are, therefore, ecologically valid decision consequences. Technologies such as augmented and virtual reality (VR) can be particularly useful in these contexts as they allow for the simulation of real-world environments within which one or more factors can be systematically manipulated (Cano Porras et al., 2018, 2019; Raffegeau et al., 2023). For example, the effects of movement context may be studied by using multiple virtual environments such as road-crossing in traffic, hiking on a trail, or navigating in a crowded mall. In combination with motion platforms or specialized treadmills that can be used to deliver the physical consequences of decisions, these virtual environments can be made to match real-world environments more closely.

While technologies such as VR and specialized treadmills can help advance the research endeavor to understand behavioral fall risk, they may not always be practical options for use in clinical practice due to financial and physical space constraints. More practical solutions for evaluating decision-making in the clinic include those similar to the plank-crossing task described previously where planks of differing levels of difficulty can be presented to walk across (Butler et al., 2015). While participants in this study did not actually cross their plank of choice due to safety concerns, future studies could implement safety measures to enable clinicians to assess their patients’ risk-taking tendencies based on which planks they choose to cross or avoid. Further work is required to develop low-cost and efficient methods to assess gait-related decision-making in the clinic.

Assessments of behavioral fall risk can also be improved by modifying assessments of fall history to better capture the circumstances surrounding a fall. These circumstances could include the individual’s goals and motivation at the time of the fall, and the exact movement or behavior that led to the fall. Although this information is prone to each of the biases associated with self-reports (Raphael, 1987), it may be used to track the conditions surrounding falls more comprehensively in both research and the clinic. Additionally, it is just as important to consider the circumstances leading to near-falls or balance disturbances, as they can provide insight into daily behaviors that put the individual in risky situations but may otherwise be overlooked.

A final challenge when translating the risky decision-making framework to locomotor control and fall risk is that of identifying ideal decisions and decision-making processes. When walking, decisions are made within the context of the individual’s intrinsic characteristics and features of their environment (Feldman and Chaudhury, 2008). These decisions then directly inform gait behavior and fall risk. Because of the many sources of sensory information available, identifying the key factors that decisions *are* based on and those that they *should be* based on is important, particularly in terms of translating this work to the clinic. Ultimately, the goal is for physical therapists to be able to recognize key features of a patient’s decision-making process and prescribe specific and actionable ways to alter it in a way that reduces fall risk. Fall risk itself is inherently multidimensional and its reliable estimation continues to be an elusive goal despite a wealth of research (Perell et al., 2001; Gates et al., 2008; Ejupi et al., 2014; Kluft et al., 2019). This is primarily because assessments of gait and balance ability at a single time point in a clinic or research setting do not entirely reflect daily behaviors in the outside world. As the use of wearable technology (Howcroft et al., 2013, 2017; Bet et al., 2019; Nouredanesh et al., 2021) for health and behavioral monitoring improves, clinicians may be able to combine data from daily gait behavior and assessments of risky decision-making to address excessive risk-seeking or risk-aversive tendencies in their patients.

## Conclusion

While it is well-established that aging and the presence of neurological impairments increase the risk of falls, much less is known about how these factors influence behavioral risk. As reviewed here, behavioral risk is influenced by an individual’s ability to assess their own capacity, the requirements of the task, the features of the environment, and the ability to integrate this information to make appropriate decisions. Impairments in any of these processes can cause impaired decision-making and when this occurs in the context of gait and balance-related movements, it can lead to potentially catastrophic outcomes. Age-related declines in physical ability are not always accompanied by a simultaneous adjustment of perceived ability, leading to higher engagement in risky behaviors (Sakurai et al., 2013; Butler et al., 2015; Liphart et al., 2016). This misjudgment of ability can stem from factors including, but not limited to, sensory impairments such as information transmission delays, increased noise, and reductions in sensitivity that affect multiple sensory modalities. The development of a rigorous computational framework to explain people’s decisions during walking may help identify the type of information that an individual uses when performing a risk assessment for walking, how the perception of this information may be distorted, and how this distortion influences the mobility-related decisions that people make in everyday life. By using VR in combination with perturbations capable of triggering losses of balance (Lee et al., 2019; Liss et al., 2022), one can create simulations of complex real-world environments to expand the current understanding of how decision-making influences fall risk in older adults and people with mobility impairments.

BOX 1 **Key points.**

- Fall risk is influenced by three types of factors—intrinsic (e.g., weakness), extrinsic (e.g., obstacles), and behavioral (e.g., risk-taking behaviors) (Connell and Wolf, 1997; Perell et al., 2001; Feldman and Chaudhury, 2008).
- We lack a systematic method to assess behavioral fall risk that does not rely on self-reports, which often do not match actual real-world behaviors.
- Aging can lead to a mismatch between actual and perceived motor ability, potentially increasing fall risk by encouraging risky motor decisions that do not align with actual ability (Butler et al., 2015, 2016; Kluft et al., 2017, 2018, 2019).
- Decision-making under risk is extensively studied in the field of behavioral economics, where risk is a function of the variability in outcomes when the same decision is repeated (Tobler and Weber, 2014).
- Computational models of decision-making used in behavioral economics have successfully explained people’s movement choices during upper extremity tasks (Tversky and Kahneman, 1992; Trommershäuser et al., 2003b; Wu et al., 2009; Delbaere et al., 2010; Grimm, 2010; Braun et al., 2011; Nagengast et al., 2011; Hak et al., 2013; O’Brien and Ahmed, 2013, 2016; Buurke et al., 2020).
- Given the success of these behavioral economic approaches in explaining upper extremity movement choices and their underlying processes, we propose that this framework can be extended to gait decisions to quantify behavioral fall risk.
- While prior studies use monetary rewards or artificial points as decision outcomes, the investigation of gait-related decisions should focus on incorporating outcomes that relate to fall risk.
- By studying how people walk in realistic virtual environments while experiencing balance disturbances such as slips and trips, we can better understand how individual differences in mobility-related decision-making influence fall risk (Lee et al., 2019; Liss et al., 2022).

BOX 2 **The description-experience gap.**

The most common form of decision-making tasks in behavioral economics involve a clear, numerical description of the possible outcomes of a decision and their associated probabilities. While such studies have provided tremendous insight into human decision-making under conditions of risk and uncertainty, such decisions are not commonplace in everyday life. These decisions which are made based on explicit descriptions are examples of “*decisions from description*.” More commonly, decisions in real life are made *from experience* in the same or similar situations. The study of such “*decisions from experience*” is relatively recent and has led to the discovery of differences in decisions based on these two modalities, commonly termed the “*description-experience gap*” (Hertwig et al., 2004; Hertwig and Erev, 2009; Wu et al., 2009; Ludvig and Spetch, 2011; Camilleri and Newell, 2013).

Early in the study of this phenomenon, it was most widely studied and reported in situations with rare events (Hertwig et al., 2004; Hertwig and Erev, 2009). The common finding is that rare events are overweighted in decisions from description but underweighted in decisions from experience. Because rare events by nature occur with low probabilities, two explanations for this finding are *limited sampling* because rare events are not experienced often in a decision from experience paradigm, and a *recency heuristic* because even if they are experienced sufficiently, more likely events may be experienced more recently and hence, receive more weight (Hertwig et al., 2004). In walking in everyday life, falls can be considered rare events, underweighting of which can lead to repeated engagement in activities that are likely to cause falls.

More recent work has found that this description-experience gap exists not only for low probability, rare outcomes but even for those with higher probabilities (Ludvig and Spetch, 2011). Specifically in the context of Prospect Theory, the common finding is that of overweighting of low probabilities and underweighting of moderate-large probabilities in decisions from description, but the opposite in decisions from experience (Wu et al., 2009; Ludvig and Spetch, 2011). In addition to the limited sampling and recency effects, a potential reason for this observed difference is that explicit probabilities are known in description-based decisions but must be inferred or learned in experience-based decisions (Hertwig and Erev, 2009; Wu et al., 2009). However, it is unclear how this difference in information format might influence probability weighting. Finally, another proposed cause of this gap is contingent sampling where in experience-based decisions, people may rely more heavily on information gained in situations similar to the current one, thereby disregarding any other important information which would otherwise be readily available in a description paradigm (Hertwig and Erev, 2009).

Most movement and gait-related decisions in everyday life are based on previous experiences. These decisions are different from lottery-based decisions from experience because of the added aspects of voluntary motor control and motor learning. It has previously been suggested that the differences seen in motor and economic decision-making, in addition to stemming from this description-experience gap, may also be due to people’s perceived ability to control the outcome of a decision when the outcome is based on their motor skill (Wu et al., 2009; O’Brien and Ahmed, 2013, 2014, 2015). Due to these differences, it is important to further investigate the description-experience gap specifically in the context of movement-related decisions.

## Author contributions

SJ: Conceptualization, Writing – original draft, Writing – review & editing, Data curation, Visualization. NS: Funding acquisition, Writing – review & editing. JF: Conceptualization, Funding acquisition, Supervision, Writing – original draft, Writing – review & editing.
